# Seasonal Dynamics Without Reset: Core Microbiota Stability Across Development in a Gall-Dwelling Weevil

**DOI:** 10.3390/insects17060544

**Published:** 2026-05-23

**Authors:** Haiming Qin, Chao Xue, Wei Li, Ran Li, Xianfeng Yi

**Affiliations:** 1 School of Life Sciences, Qufu Normal University, Qufu 273165, China; 2 Mingguangsi Forest Farm, Pingyi County, Linyi 273300, China

**Keywords:** *Coccotorus beijingensis*, core microbiota, seasonal succession, larval development, functional prediction

## Abstract

*Coccotorus beijingensis* is a typical gall-inducing insect whose larvae complete their development within enclosed galls, providing a unique model for investigating host–microbe symbiosis. This study aimed to characterize the dynamic succession of the symbiotic microbiota in *C. beijingensis* across the larval stage (April–August) and pupal stage (September). Using high-throughput 16S rRNA gene sequencing, we performed a systematic analysis of monthly collected samples spanning the larval and pupal stages. The results revealed significant temporal variation in the structure of the larval microbial community. Bacillota and Bacteroidota were the dominant bacterial phyla throughout development. Although non-core bacterial groups, such as Pseudomonadota, showed pronounced seasonal fluctuations, no clear microbial reset was observed during metamorphosis. Exploratory PICRUSt2-based functional prediction suggested that the predicted metabolic potential of the microbiota may vary across development, with pathways related to carbohydrate metabolism, amino acid metabolism, and energy metabolism showing higher predicted representation during the middle larval stages. Overall, this study demonstrates that, within the confined gall microhabitat, larval development and seasonal dynamics jointly drive the temporal restructuring and functional adaptation of the microbial community. These findings provide new insights into the symbiotic mechanisms of holometabolous insects and their associated microbiota.

## Simple Summary

Insects often depend on associated bacteria for nutrition, development, and environmental adaptation, but these communities may change during growth. We studied this process in *Coccotorus beijingensis*, a gall-dwelling weevil whose larvae and pupae develop inside enclosed plant tissue. We found that the bacterial community changed across months and developmental stages, but its core members remained stable. A dominant symbiotic bacterium persisted throughout development, whereas other bacterial groups varied seasonally. Unlike some other holometabolous insects, this species showed no clear microbial reset during metamorphosis. These results improve our understanding of insect–microbe interactions and suggest that the closed gall environment may help maintain a stable core microbiota, with potential value for future pest management.

## 1. Introduction

The symbiotic microbiota of insects forms complex and intimate associations with their hosts, exerting profound effects on host physiology, ecology, and evolutionary adaptation [1]. These microbial communities contribute to a wide range of essential functions, including nutrient provisioning, development, immune regulation, and protection against pathogens, reflecting long-term co-evolutionary interactions between insects and their associated microorganisms [2,3,4,5]. In many insect lineages, microbial symbionts are indispensable for host survival and ecological specialization. For example, the gut microbiota of termites and cockroaches mediates the degradation of recalcitrant lignocellulose, thereby enabling the utilization of wood and humus as major nutritional resources and supporting normal growth and development [6]. Likewise, aphids depend on obligate endosymbionts such as *Buchnera aphidicola* to synthesize essential amino acids absent from their phloem-based diet [7]. Beyond nutritional support, insect-associated microbiota can also shape key behavioral and physiological traits, including olfactory responses and thermal tolerance [8,9].

Insect microbial communities are not static but instead undergo dynamic succession over the course of host development. Such variation is shaped by the interplay of intrinsic factors, including developmental stage, immune status, and molting, and extrinsic factors such as diet, environmental exposure, and social interactions. In holometabolous insects in particular, distinct life stages often occupy different ecological niches and exhibit markedly different physiological demands, frequently resulting in substantial microbial community restructuring. For instance, the gut microbiota of *Apis mellifera* is acquired after eclosion from the hive environment, whereas the pupal stage of *Aedes aegypti* acts as an ecological filter that influences the composition of the adult microbiota [10]. These observations support the view that holometabolous development constitutes a critical window for microbial community reassembly and functional reorganization.

The family Curculionidae, with more than 60,000 described species, represents one of the most evolutionarily successful radiations in insects, and its remarkable ecological diversification is widely considered to be closely associated with symbiotic interactions that alleviate the nutritional constraints imposed by specialized feeding habits [11]. Within this lineage, gall-inducing weevils provide an especially valuable system for investigating host–microbe symbiosis because of their distinctive tripartite interaction involving insect, host plant, and gall [12]. *Coccotorus beijingensis*, an obligate parasite of *Celtis bungeana*, completes its entire life cycle within enclosed galls and exhibits strict developmental synchrony with its host plant. This highly specialized lifestyle offers an unusual opportunity to explore microbial dynamics in a relatively isolated and environmentally buffered microhabitat. Although previous studies have examined physiological and molecular aspects of this insect–plant–gall system, a comprehensive characterization of the microbial community associated with *C. beijingensis* across its life cycle remains unavailable. Existing research has focused primarily on particular bacterial symbionts or on the adult gut microbiota, while largely overlooking temporal microbial succession across developmental stages [13]. This gap limits our understanding of how microbial community dynamics during holometabolous metamorphosis contribute to host adaptation in such a specialized ecological context.

In this study, we investigated the seasonal dynamics of the symbiotic bacterial community of *C. beijingensis* from spring to autumn to determine how developmental progression influences microbiota composition and stability. We hypothesized that, unlike insects developing in open environments, where microbial communities are frequently disrupted by environmental exchange, the stable and enclosed conditions within galls facilitate the persistence of a core microbiota throughout metamorphosis. The physical isolation and relatively stable microclimate of the gall are expected to reduce external microbial inputs and favor trans-stadial persistence or long-term maintenance of specific symbiotic taxa across developmental stages. At the same time, seasonal changes in nutrient availability, such as variation in host plant sap composition or gall tissue chemistry during the growing season, may drive predictable fluctuations in non-core microbial taxa. These transient microbial members may increase under nutrient-rich conditions and decline as resources become limited, indicating a stronger dependence on changing environmental conditions than on stable integration into the host-associated symbiotic network. To test these hypotheses, we conducted a systematic analysis of bacterial communities in 58 samples collected across spring, summer, and autumn. Samples were obtained through a six-month field monitoring program from populations inhabiting sites standardized for latitude, altitude, and ecological conditions and were subsequently analyzed using high-throughput 16S rRNA gene sequencing. Our results revealed a consistent temporal succession pattern in the microbial community and identified key indicator taxa associated with seasonal shifts. These findings deepen our understanding of host–microbe interactions in confined ecological niches and provide a basis for the development of microbiome-informed strategies for managing this gall-inducing pest.

## 2. Materials and Methods

### 2.1. Sample Collection

**Figure 1:**
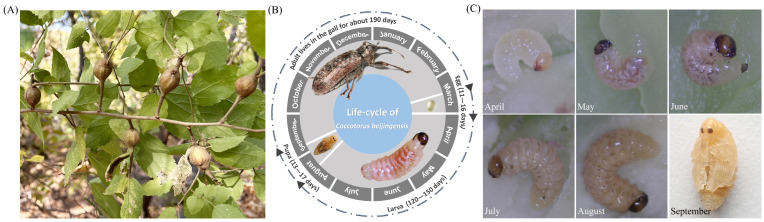
Gall morphology, life cycle diagram, and morphological transition of *C. beijingensis*. Morphological characteristics of the galls induced by *C. beijingensis* (**A**); life cycle diagram of *C. beijingensis* in the Shimenshan area (**B**); and morphological transition of *C. beijingensis* from the larval to the pupal stage (**C**).

*Coccotorus beijingensis* is an obligate parasite of *Celtis bungeana* and induces spherical galls on the twigs of its host plant (Figure 1A). Its entire life cycle is completed within these enclosed galls (Figure 1B), where both larval and pupal development occur. Although adults emerge by gnawing a small exit hole in the gall wall, they remain inside the galls for an extended period because of low temperatures until the following March. Therefore, this enclosed gall system provides an ideal model for investigating how the developmental stage influences the symbiotic microbiota of *C. beijingensis*. In this study, larval and pupal individuals collected in different months exhibited distinct stage-specific morphological characteristics (Figure 1C). These traits not only clearly reflect developmental progression but also provide a direct morphological basis for evaluating stage consistency during sampling, thereby improving sampling accuracy and the reliability of subsequent analyses of the relationship between symbiotic microbial succession and host development.

This study was conducted in Shimenshan National Forest Park, Qufu City, Shandong Province, China (35°38′27.6″ N, 117°07′08.4″ E), from April to September 2023. Eight biological replicates were collected in April, whereas ten biological replicates were collected in each of the remaining months. Each biological replicate consisted of a pooled sample of 50–60 individuals collected from multiple dissected galls within the same sampling month. To minimize potential contamination by exogenous microorganisms, galls were dissected under sterile conditions, and *C. beijingensis* individuals were collected using sterilized forceps. All samples were immediately transferred into sterile centrifuge tubes, rapidly frozen in liquid nitrogen at the collection site to preserve microbial community integrity, and subsequently stored in an ultra-low-temperature freezer until DNA extraction.

### 2.2. DNA Extraction and 16S rRNA Gene Amplification

Total genomic DNA was extracted from *C. beijingensis* individuals using the MagPure Soil DNA LQ Kit (Magen, Guangzhou, China) according to the manufacturer’s instructions. DNA concentration and integrity were assessed using a NanoDrop 2000 spectrophotometer (Thermo Fisher Scientific, Waltham, MA, USA) and agarose gel electrophoresis, respectively. The V3–V4 hypervariable region of the bacterial 16S rRNA gene was amplified using primers 343F (5′-TACGGRAGGCAGCAG-3′) and 798R (5′-AGGGTATCTAATCCT-3′). PCR amplification was performed in a 25 μL reaction mixture containing 10 ng of template DNA, 12.5 μL of 2× Gflex PCR Buffer, 0.5 μL of Tks Gflex DNA Polymerase, 1.0 μL of each primer (10 μM), and nuclease-free water to a final volume of 25 μL, according to the manufacturer’s protocol with minor modifications. The PCR cycling conditions were as follows: initial denaturation at 94 °C for 1 min; 30 cycles of denaturation at 98 °C for 10 s, annealing at 60 °C for 15 s, and extension at 68 °C for 30 s; followed by a final extension at 68 °C for 5 min.

### 2.3. Library Construction

After verification by gel electrophoresis, PCR amplicons were purified using AMPure XP beads (Beckman Coulter, Brea, CA, USA). The purified products were quantified using the Qubit dsDNA HS Assay Kit (Thermo Fisher Scientific, Waltham, MA, USA) and pooled at equimolar concentrations to construct the sequencing library. Library quality was assessed using an Agilent 2100 Bioanalyzer (Agilent Technologies, Santa Clara, CA, USA). Final sequencing was carried out on an Illumina MiSeq platform (Illumina, San Diego, CA, USA) using a paired-end 2 × 250 bp strategy. All library construction and sequencing procedures were performed by OE Biotech Co., Ltd. (Shanghai, China).

### 2.4. Bioinformatic Analysis of Sequencing Data

Raw reads generated from Illumina sequencing were processed in FASTQ format for quality control and downstream bioinformatic analysis. Adapter and primer sequences were trimmed, and reads containing ambiguous bases or low-quality bases were removed using Trimmomatic [14]. High-quality paired-end reads were subsequently merged using FLASH [15] according to overlap information. The filtered reads were further processed in QIIME2 (v2021.11) using the DADA2 plugin [16,17], which performs quality filtering, denoising, paired-end merging, and chimera removal to infer amplicon sequence variants (ASVs) at single-nucleotide resolution. Representative ASV sequences were then obtained for taxonomic assignment and community analysis.

Taxonomic classification was carried out using the RDP classifier with a confidence threshold of 70% [18]. Representative sequences were annotated against the SILVA 132 reference database [19] to determine bacterial taxonomic identities. Because *Wolbachia* was overwhelmingly dominant and represents an intracellular endosymbiont rather than a typical extracellular gut-associated bacterium, the ASV assigned to *Wolbachia* was removed before downstream analyses of taxonomic composition, differential taxa, and functional prediction. The remaining non-Wolbachia bacterial community was then used to evaluate seasonal turnover and developmental changes in the associated microbiota. Sequencing depth and ASV summary statistics were calculated before Wolbachia removal, whereas downstream analyses of alpha diversity, beta diversity, taxonomic composition, differential taxa, and functional prediction were performed using the non-Wolbachia bacterial community.

### 2.5. Data Analysis

All statistical and microbial community analyses were performed in R v4.1.2 using the phyloseq v1.38.0, vegan v2.5-7, and related packages [20], implemented on the bioinformatics cloud platform provided by OE Biotech Co., Ltd. (https://cloud.oebiotech.com; accessed on 20 November 2025). Alpha diversity was estimated using the Chao1, ACE, Shannon, and Simpson indices, and differences among groups were assessed using the Kruskal–Wallis test. Beta diversity was calculated based on Bray–Curtis dissimilarity and unweighted UniFrac distances and was visualized by principal coordinates analysis (PCoA) and non-metric multidimensional scaling (NMDS). Unweighted UniFrac distances were calculated based on a phylogenetic tree constructed from representative ASV sequences and were used to assess phylogenetically informed differences in microbial community composition among samples. Permutational multivariate analysis of variance (PERMANOVA), implemented using the adonis function, was used to test for significant differences in community composition among months. Differentially abundant taxa were identified using the Kruskal–Wallis test with false discovery rate (FDR) correction. Linear discriminant analysis effect size (LEfSe) analysis was further performed to screen for potential biomarker taxa among months using an LDA score threshold of >3.0 and a significance threshold of *p* < 0.05. Core microbiota were defined as ASVs detected in all biological replicates within a given group and with a relative abundance of ≥0.01%. Functional prediction of microbial communities was conducted using PICRUSt2 v2.5.2, and KEGG pathway profiles were compared to assess potential functional changes across developmental stages.

## 3. Results

### 3.1. Temporal Dynamics of the Symbiotic Microbiota in C. beijingensis

After quality filtering, chimera removal, and decontamination, a total of 4,269,866 high-quality 16S rRNA gene sequences were obtained from 58 samples, with an average of 73,618 ± 2358 sequences per sample. DADA2 analysis yielded 5473 amplicon sequence variants (ASVs) (Table S1). Among these, 290 ASVs were unique to April, 1157 to May, 1101 to June, 1387 to July, 1534 to August, and 979 to September (Figure S1; Table S2). Unless otherwise stated, subsequent diversity, taxonomic, differential abundance, and functional analyses were conducted after removing Wolbachia-assigned ASVs.

Rarefaction curves based on the Chao1, Shannon, and PD whole-tree indices, together with rank–abundance curves, indicated that sequencing depth was sufficient to capture the diversity of the non-Wolbachia bacterial community in all samples (Figures S2 and S3). In addition, Good’s coverage exceeded 99.9% for all samples (Table S1), further confirming adequate sequencing depth. Alpha-diversity analyses showed significant differences in community richness between April and each of the subsequent months, including May, June, July, August, and September (Figure S4; Table S1).

**Figure 2:**
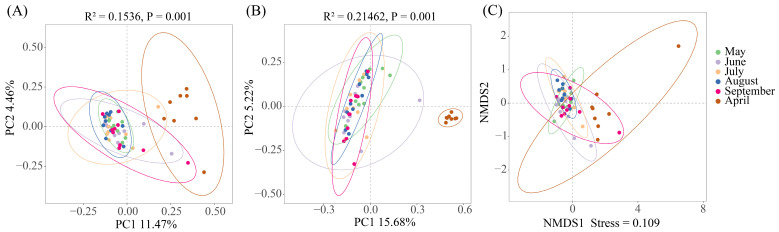
Beta diversity of the non-*Wolbachia* bacterial community of *C. beijingensis* across developmental stages. Principal coordinate analysis (PCoA) based on unweighted UniFrac distances (**A**) and Bray–Curtis distances (**B**), and non-metric multidimensional scaling (NMDS) analysis (**C**). The horizontal and vertical axes represent the first two ordination components, and the percentages indicate the proportion of variation explained by each component. Distances between sample points reflect differences in community composition. Points of different colors or shapes represent samples from different groups.

Beta-diversity analyses further revealed clear temporal variation in the symbiotic bacterial communities of *C. beijingensis*. Principal coordinate analysis (PCoA) based on unweighted UniFrac distances showed significant separation among months (PERMANOVA/adonis, *R*^2^ = 0.1536, *p* = 0.001; Figure 2A), and a similar pattern was observed for PCoA based on Bray–Curtis distances (PERMANOVA/adonis, *R*^2^ = 0.21462, *p* = 0.001; Figure 2B). Notably, samples collected in April were significantly differentiated from those obtained in all other months (Table S3), as confirmed by post hoc pairwise PERMANOVA tests. NMDS ordination also showed month-dependent variation in community composition, with samples from different months tending to cluster separately in two-dimensional space (Figure 2C).

### 3.2. Composition and Temporal Succession of the Microbial Community

**Figure 3:**
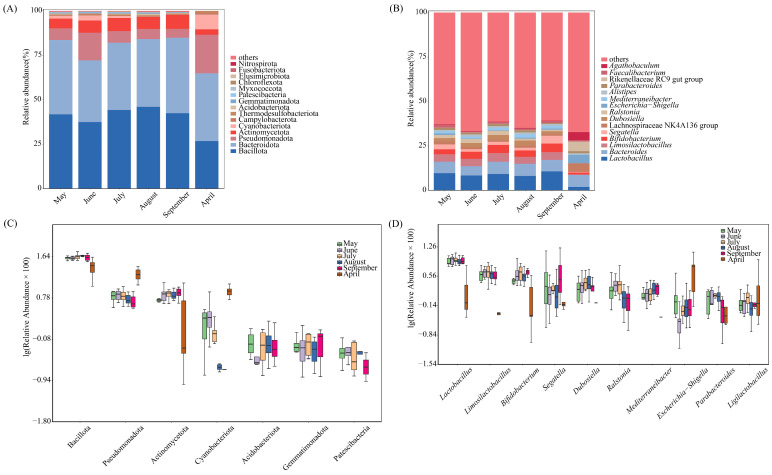
Relative abundance of non-Wolbachia bacterial taxa at the phylum level (**A**) and genus level (**B**) across developmental stages (top 15 most abundant taxa). Box plots comparing the top taxa showing significant temporal variation at the phylum (**C**) and genus (**D**) levels.

To better characterize seasonal turnover in the non-endosymbiotic bacterial community, the dominant intracellular endosymbiont *Wolbachia* was excluded from the following taxonomic composition analyses. Taxonomic annotation against the SILVA database revealed marked temporal variation in the relative abundance of major bacterial taxa during development from the larval stage (April–August) to the pupal stage (September) (Figure 3A,B). At the phylum level, Bacillota exhibited significant temporal fluctuations, whereas Bacteroidota remained relatively stable throughout development.

Bacillota showed low abundance in April (26.79%), increased to an intermediate level in June (37.48%), peaked in August (46.01%) (*p* = 0.0008), and then changed thereafter. For Bacteroidota, relative abundances ranged from a minimum of 37.84% in July to a maximum of 42.46% in September; however, these temporal variations were not statistically significant (*p* > 0.05). The relative abundance of Pseudomonadota peaked in April (21.6%), declined in May (6.55%), and then increased again to 15.55% in June but decreased to the lowest level in September (4.97%) (*p* = 0.0002). Actinomycetota remained at low abundance overall (<10%) but showed a distinct minor peak in September (7.89%) (*p* = 0.0046). Specifically, Cyanobacteriota remained much higher in April compared to other months (*p* < 0.001). Other phyla, including Acidobacteriota, Gemmatimonadota, and Patescibacteria, each accounted for less than 0.1% of the total community. Kruskal–Wallis tests indicated significant differences in the relative abundance of these minor bacterial phyla across months (*p* = 0.001, *p* = 0.032, and *p* = 0.049), indicating pronounced seasonal and developmental succession in the symbiotic microbiota of *C. beijingensis* (Figure 3C).

At the lower taxonomic level, Kruskal–Wallis tests confirmed unclassified_Muribaculaceae as the predominant taxon throughout the entire sampling period. Its abundance was lowest in April (12.13%), increased to 26.77% in May, and recovered to 27.71% in September after a decline in June, July, and August (*p* = 0.018). In contrast, the relative abundances of the remaining dominant taxa were generally low, although most reached their highest values in July before gradually declining thereafter. Among these, *Bacteroides* remained stable throughout the larval development (*p* > 0.05). In contrast, *Lactobacillus* was least abundant in April (1.95%), increased in May (9.69%), and peaked in September (10.69%), showing a marginally significant difference (*p* = 0.084). Other taxa, such as *Limosilactobacillus* and *Bifidobacterium*, reached peak abundance in July or September and showed varying degrees of increase relative to April (*p* = 0.0014, *p* = 0.0418).

To further visualize temporal patterns in microbial composition, a heatmap of dominant taxa was generated for samples collected from April to September (Figure S5), showing clear month-dependent shifts in the abundance of gut community members.

### 3.3. Development-Associated Biomarkers of the Microbial Community

**Figure 4:**
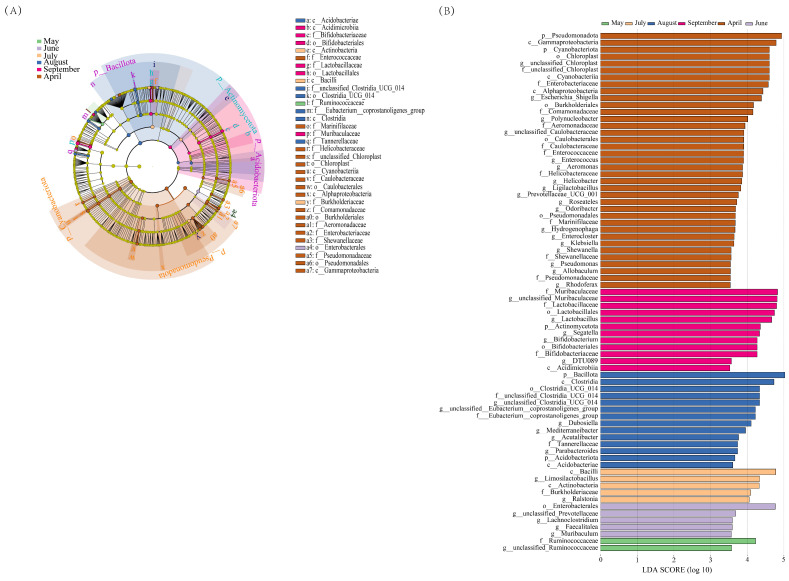
Linear discriminant analysis effect size (LEfSe) of microbial abundance across developmental stages of *C. beijingensis*: cladogram of microbial communities (**A**); differentially abundant microbial biomarkers identified by LEfSe (**B**).

To identify bacterial taxa associated with different developmental periods of *C. beijingensis*, we performed linear discriminant analysis effect size (LEfSe) analysis (Figure 4). Based on the selected threshold (LDA score > 3.0, *p* < 0.05), several taxa showed month-associated variation in relative abundance. In April, Pseudomonadota, Cyanobacteriota, Gammaproteobacteria, Cyanobacteriia, Alphaproteobacteria, Enterobacteriaceae, and *Escherichia-Shigella* were identified as potential indicator taxa. Enterobacterales was associated mainly with June. Burkholderiaceae, Actinobacteria, Bacilli, *Ralstonia*, and *Limosilactobacillus* were identified as potential indicator taxa associated with July. Bacillota, Clostridia, Clostridia_UCG_014, unclassified_Clostridia_UCG_014, *Dubosiella*, and unclassified_Eubacterium_coprostanoligenes_group were associated with August. In September, Actinomycetota, Bifidobacteriales, Lactobacillales, Bifidobacteriaceae, Lactobacillaceae, Muribaculaceae, *Bifidobacterium*, *Lactobacillus*, and unclassified_Muribaculaceae were identified as potential indicator taxa. Collectively, these results suggest month-dependent shifts in the relative abundance of several bacterial taxa during the development of *C. beijingensis*. Because LEfSe and Kruskal–Wallis tests mainly identify overall differences among groups, these taxa were interpreted as potential biomarkers rather than definitive month-specific enriched taxa unless supported by pairwise comparisons.

### 3.4. Exploratory Functional Prediction of the Microbiota

**Figure 5:**
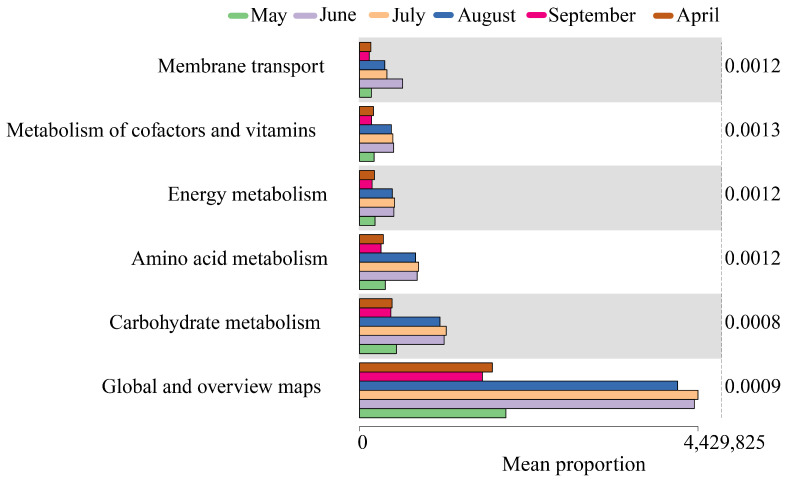
Predicted functions of bacterial communities associated with *C. beijingensis*. Representative predicted KEGG level 2 pathways showing temporal variation are shown and grouped into major functional categories.

PICRUSt2-based functional prediction was used as an exploratory approach to infer the potential functional profiles of the bacterial community associated with *C. beijingensis* from April to September (Figure 5). Because this approach relies on phylogenetic inference from reference genomes, the results should be interpreted as predicted functional potential rather than direct measurements of microbial activity. At KEGG level 2, Kruskal–Wallis tests showed that the predicted functional profiles varied temporally in pathways related to carbohydrate metabolism (*p* = 0.0008), amino acid metabolism (*p* = 0.001), energy metabolism (*p* = 0.001), metabolism of cofactors and vitamins (*p* = 0.001), and membrane transport (*p* = 0.001). These pathways showed higher predicted representation in June, July, and August than in April, May, and September (Figure 5). These predicted patterns suggest that the functional potential of the non-*Wolbachia* bacterial community may change during host development, but further metagenomic, transcriptomic, or metabolomic validation is required.

## 4. Discussion

Holometabolous development produces profound morphological and physiological differences among life stages, creating both challenges and opportunities for associated microbial communities. In the present study, we demonstrated that the symbiotic microbiota of *C. beijingensis* undergoes a clear temporal succession from the larval to the pupal stage and is jointly shaped by host development and seasonal progression. Although this general pattern is consistent with the dynamic microbial restructuring reported in many holometabolous insects [21], the lifelong confinement of *C. beijingensis* within enclosed galls results in a distinct successional trajectory compared with insects developing in more exposed environments. In particular, although the core bacterial group of the Bacillota, rather than Bacteroidota, exhibited more pronounced seasonal fluctuations among the dominant core phyla, no evidence of a marked microbial reset was detected during pupation.

### 4.1. Constraints and Selection Mechanisms Imposed by Development and Seasonality

The larval–pupal development and seasonal progression create a strong bottleneck effect and selective pressure on the microbiota of *C. beijingensis*. Microbial diversity and abundance fluctuated significantly over six months, aligning with the ‘habitat modification’ concept and known seasonal dynamics in herbivorous insects [22,23]. Both developmental stages occur within galls, where shifting nutrient composition imposes intense microhabitat-driven selection [24]. Diversity was lowest in April, and the Kruskal–Wallis tests confirmed significant differences in community richness between April and each subsequent month, consistent with seasonal fluctuation patterns [25].

Bacillota and Bacteroidota remained two dominant phyla throughout development and displayed contrasting patterns: a rapid increase after May in Bacillota, while Bacteroidota remained stable across development stages. These patterns support their status as core bacterial phyla in this system [26]. Within Bacteroidota, unclassified members of the family Muribaculaceae represented the predominant lower-level taxon and remained at relatively high abundance throughout development, which is consistent with their widespread and often functionally important association with arthropods [27,28]. In contrast to honeybees, in which pupation is often accompanied by a pronounced restructuring or reset of the microbiota [2], the enclosed gall habitat of *C. beijingensis* likely limits external microbial input and reduces environmentally driven recolonization. As a result, microbial variation in this species appears to be governed primarily by internal developmental and seasonal processes rather than by repeated exchange with the surrounding environment. This pattern differs markedly from that of insects such as *Blattella germanica*, which experience continuous microbial acquisition from external sources across development [29].

As a specialist herbivore dependent on a single host plant and a highly restricted nutrient source, *C. beijingensis* may rely more strongly on stable core symbionts than polyphagous or oligophagous insects, whose microbial communities often shift substantially in response to diet variation [30]. This observation is consistent with the idea that holometabolous insects can maintain persistent symbionts with specific adaptive value despite developmental transitions [22]. In this context, Bacillota, Bacteroidota, and other core bacteria may persist by virtue of long-term host association and specialized compatibility with the gall environment, representing a stable symbiotic module that buffers developmental and seasonal perturbations [31]. The enclosed gall microhabitat likely restricts dispersal and promotes the retention of host-associated symbionts, as has also been suggested for other physically isolated insect-associated microbial systems [32].

Interestingly, the relative abundance of Pseudomonadota declined temporarily after April, coinciding with increases in Bacillota, suggesting a transient relaxation of selective constraints. This shift may reflect changes in nutrient availability, host physiology, or immune regulation during mid-season development. Such a pattern suggests a balance between the persistence of stable core symbionts and the temporary expansion of facultative or opportunistic taxa [22]. Unlike *Drosophila*, in which non-core microbes may be actively reduced during metamorphosis [33], *C. beijingensis* showed no evidence of a comparable microbial reset during pupation. Instead, the increase in non-core bacteria in July appears more likely to reflect nutrient-driven proliferation within the enclosed gall habitat, followed by a decline in Pseudomonadota and a relative increase in taxa such as Lactobacillus, Bifidobacterium, and unclassified_Muribaculaceae during the pupal stage.

### 4.2. Developmental and Seasonal Drivers of Stage-Specific Symbiotic Flexibility

The microbial succession observed in *C. beijingensis* suggests that holometabolous development and seasonality together generate stage-specific flexibility in host–microbe interactions. This pattern is broadly consistent with the concept of adaptive decoupling, whereby different developmental stages optimize physiological traits and symbiotic associations to meet distinct ecological and metabolic demands [22]. However, in *C. beijingensis*, this transition occurs without an obvious microbial reset during pupation, distinguishing it from insects developing in exposed environments, where metamorphosis is often associated with more extensive microbial turnover [33,34].

A major driver of this flexibility is likely seasonal variation in the nutritional and chemical properties of gall tissues. Even during the non-feeding pupal stage, the host remains embedded within gall tissues, and residual resources or stored metabolites may continue to influence microbial community function. Functional prediction analyses indicated that the metabolic potential of the microbiota shifted across development in association with these changing conditions. During the middle to late larval stages (June–August), pathways related to carbohydrate metabolism, amino acid metabolism, and energy metabolism showed higher predicted representation, suggesting that the microbiota may support rapid larval growth under nutrient-demanding conditions [7]. In contrast, during the pupal stage (September), the above pathways became depleted, which may reflect decreased requirements for developmental remodeling in response to seasonal changes in gall carbohydrates [35,36].

This pattern contrasts with systems such as honeybees, in which microbial function is strongly influenced by diverse dietary sources and frequent environmental exposure [2,19]. In *C. beijingensis*, by contrast, larvae feed exclusively on gall tissue, and pupae do not feed, meaning that microbial functional dynamics are likely governed primarily by changes in host physiology and in the internal gall environment. Under such conditions, the plasticity of core symbionts may be especially important for maintaining developmental homeostasis.

Temporal fluctuations in dominant and accessory taxa, including unclassified members of the family Muribaculaceae and genera such as *Lactobacillus* and *Limosilactobacillus*, particularly during July and September, further support the existence of limited but ecologically meaningful symbiotic flexibility. These taxa may provide supplementary metabolic functions during periods of elevated physiological demand. Although the gall is physically enclosed, seasonal changes in temperature, humidity, and host plant exudates may still permit rare and transient acquisition of environmentally derived bacteria [37]. The LEfSe analysis identified Enterobacterales as a biomarker in June, which may reflect such a process [38]. Rather than indicating a complete restructuring of the microbiota, these seasonal additions may represent opportunistic and temporary integrations superimposed on a stable core microbiota. Thus, the persistence of core symbionts in *C. beijingensis* appears to depend more on the protective and filtering properties of the gall microhabitat than on active elimination of transient taxa by host immune processes.

### 4.3. Functional Significance of Microbial Regulation for Larval Development

The larval development of *C. beijingensis* is likely supported by stage-specific functional contributions from its associated microbiota. Functional prediction indicated that the middle to late larval stages, particularly June to August, showed higher predicted representation of pathways related to carbohydrate metabolism, amino acid metabolism, energy metabolism, and metabolism of cofactors and vitamins. These patterns suggest that the microbiota may contribute to nutrient transformation and energy metabolism during periods of rapid larval growth. Such nutritional supplementation is a common feature of phytophagous insect–microbe symbioses [7]. In addition, non-core taxa, including Pseudomonadota and Cyanobacteriota and genera such as *Bacteroides* and *Limosilactobacillus*, may provide transient metabolic complementation during specific developmental periods.

This symbiotic system appears to be highly specialized. Unlike honeybees, in which multiple gut taxa contribute cooperatively to nutrition and microbial functions shift extensively after pupation in association with diet change [2,29], *C. beijingensis* seems to rely predominantly on Muribaculaceae and other core symbionts (such as *Bacteroides* and *Lactobacillus*) for functional stability, whereas non-core microbes provide only transient metabolic complementation. Muribaculaceae is widely recognized as a bacterial family associated with the breakdown and utilization of complex carbohydrates [39,40]. Its relatively high abundance across developmental stages, together with the higher predicted representation of carbohydrate metabolism pathways during June to August, suggests a potential role in carbohydrate utilization during larval development. Likewise, the temporary increase in Actinobacteriota observed in September may also be functionally relevant, because members of this group are well known for producing antimicrobial compounds [41].

Seasonal variation in these potentially defensive taxa suggests that immune-related microbial functions may also be dynamically regulated across development, as reported in other symbiotic systems such as aphids and social insects [42,43,44]. Nevertheless, the functional roles inferred here remain predictive rather than experimentally validated. Future studies integrating metagenomics, metabolomics, and direct pathogen challenge assays will be necessary to determine whether the dominant symbionts of *C. beijingensis* directly mediate nutrient provisioning, detoxification, or pathogen resistance [45,46,47]. It should be noted that the PICRUSt2 results represent predicted functional potential rather than direct functional activity. Because current reference databases underrepresent many specialized insect-associated bacteria, especially lineages with reduced genomes or poorly characterized functions, these results should be interpreted cautiously. Future studies integrating shotgun metagenomics, metatranscriptomics, metabolomics, and experimental validation will be needed to confirm the functional roles of the dominant bacterial taxa associated with *C. beijingensis*.

## 5. Conclusions

This study systematically characterized the symbiotic microbiota of *C. beijingensis* during the larval–pupal transition and demonstrated a distinctive pattern of microbial succession within the enclosed gall microhabitat. Compared with holometabolous insects developing in exposed environments, the microbiota of *C. beijingensis* exhibited three major characteristics: no clear microbial reset occurred during pupation; the core bacterial phyla Bacillota and Bacteroidota persisted throughout development; and microbial succession was driven mainly by host developmental stage and seasonal dynamics, with limited influence from external microbial colonization. Furthermore, the functional stability of the microbial community was primarily maintained by core symbionts, whereas non-core taxa displayed temporal variation and likely contributed auxiliary functions during particular developmental stages. Collectively, these results emphasize the importance of microhabitat-mediated selection in shaping the composition and function of insect-associated microbiota. They further suggest that the enclosed gall environment may profoundly influence microbiota assembly and stability in holometabolous insects. This study enriches our understanding of host–microbe symbiosis in gall-inducing insects, provides new insights into the adaptive divergence of insect microbiota across ecological contexts, and offers a theoretical foundation for future microbiome-based management strategies targeting gall weevils.

## Data Availability

The original contributions presented in this study are included in the article/Supplementary Materials. Further inquiries can be directed to the corresponding authors.

## Supplementary Materials

The following supporting information can be downloaded at: https://www.mdpi.com/article/10.3390/insects17060544/s1, Figure S1: Venn diagram showing the distribution of amplicon sequence variants (ASVs) among symbiotic microbiota across developmental stages of *C. beijingensis*. Figure S2: Rarefaction curves of the 16S rRNA gene reads based on inferred ASVs at 99% sequence similarity. (A) Chao1; (B) Shannon; (C) PD whole-tree index. Figure S3: Rank–abundance curve showing the distribution of microbial taxa across different sampling months. Figure S4: Alpha diversity of the symbiotic microbiota across developmental stages of *C. beijingensis*. (A) ACE; (B) Chao1; (C) Shannon; (D) Simpson. Figure S5: Heatmap of dominant taxa across developmental stages of *C. beijingensis*. The color scale represents relative abundance, ranging from blue (low abundance) to red (high abundance). Table S1: Sequencing analysis of 16S rRNA gene amplicons of *C. beijingensis* with diversity indices. Table S2: Amplicon sequence variant (ASV) counts at six taxonomic levels for each sample. Table S3: Pairwise PERMANOVA (adonis2) tests of beta-diversity distances.
