# Supplementary material for: Seasonal Dynamics Without Reset: Core Microbiota Stability Across Development in a Gall-Dwelling Weevil
Source: Insects. 2026 May 23;17(6):544. doi: 10.3390/insects17060544 (PMC13299049; doi:10.3390/insects17060544)
Supplement: Supplementary file 1 [file insects-17-00544-s001.zip › Figure S.pdf]

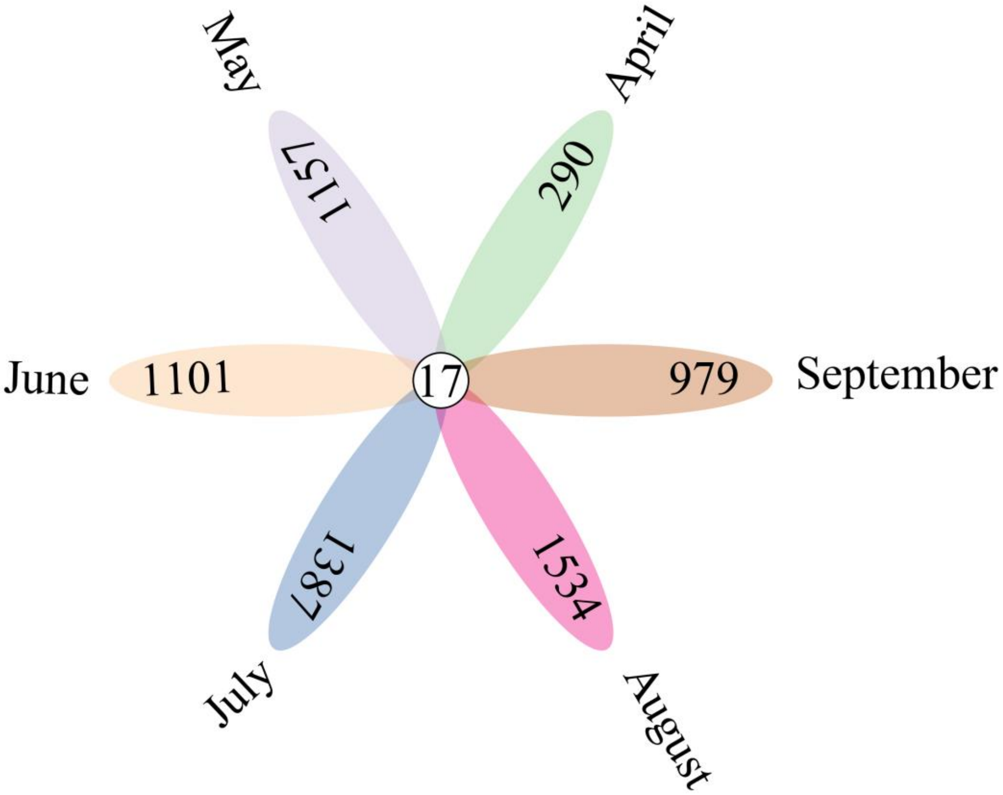

Figure S1 Venn diagram showing the distribution of amplicon sequence variants (ASVs) among symbiotic microbiota across developmental stages of *C. beijingensis*.

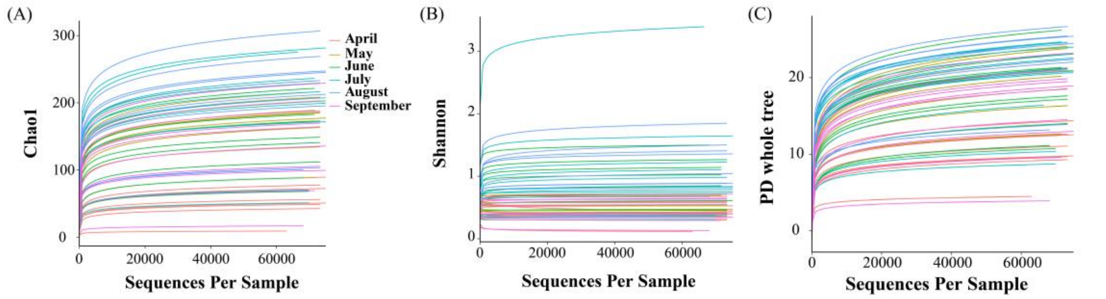

Figure S2 Rarefaction curves of the 16S rRNA gene reads based on ASVs at 99% sequence similarity. (A) Chao1; (B) Shannon; (C) PD whole-tree index.

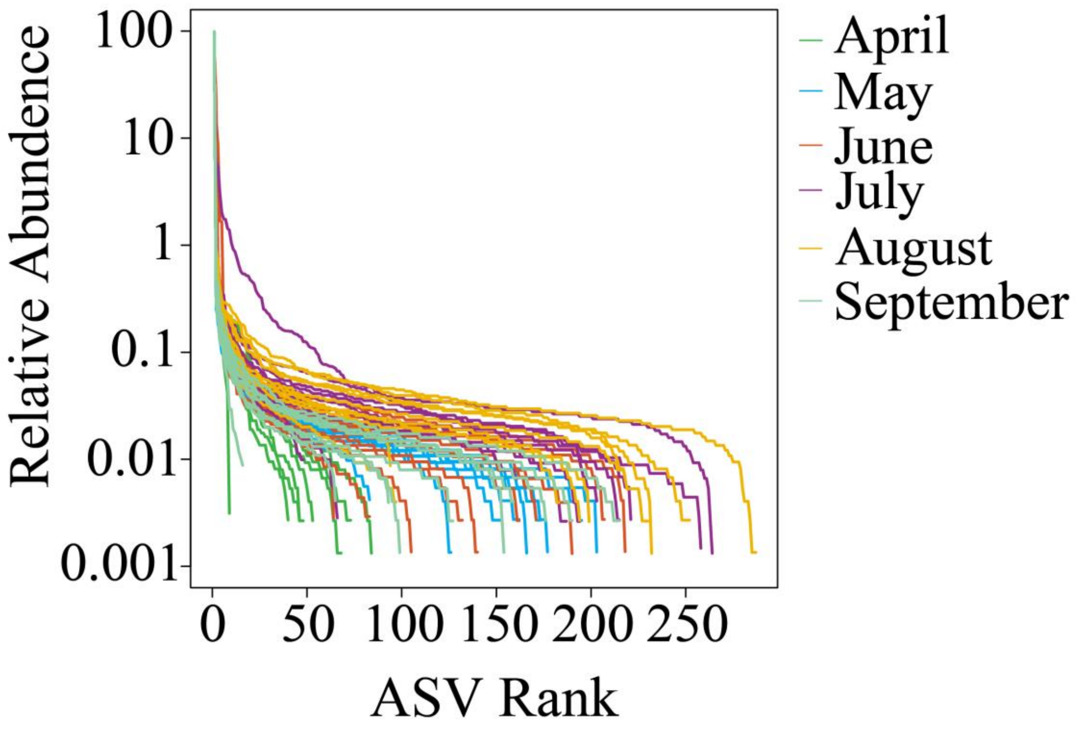

Figure S3 Rank-abundance curve showing the distribution of microbial taxa across different sampling months.

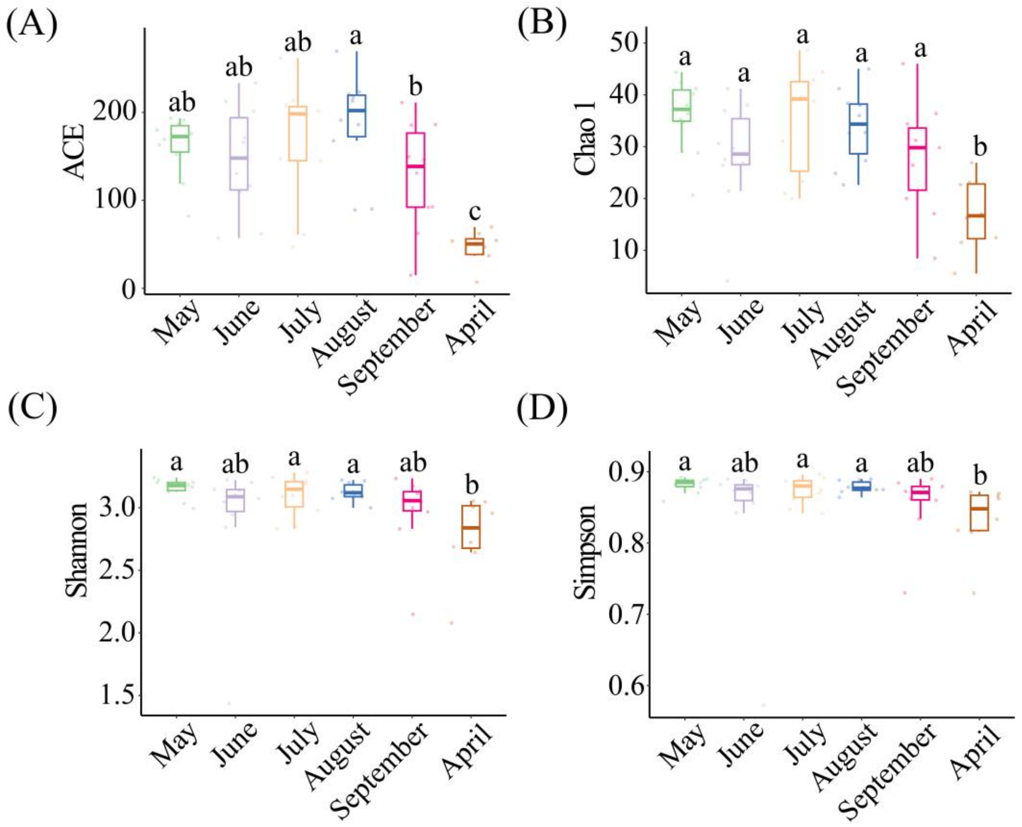

Figure S4 Alpha diversity of symbiont microbiota across the larval stage of *C. beijingensis*. (A) ACE; (B) Chao1; (C) Shannon; (D) Simpson.

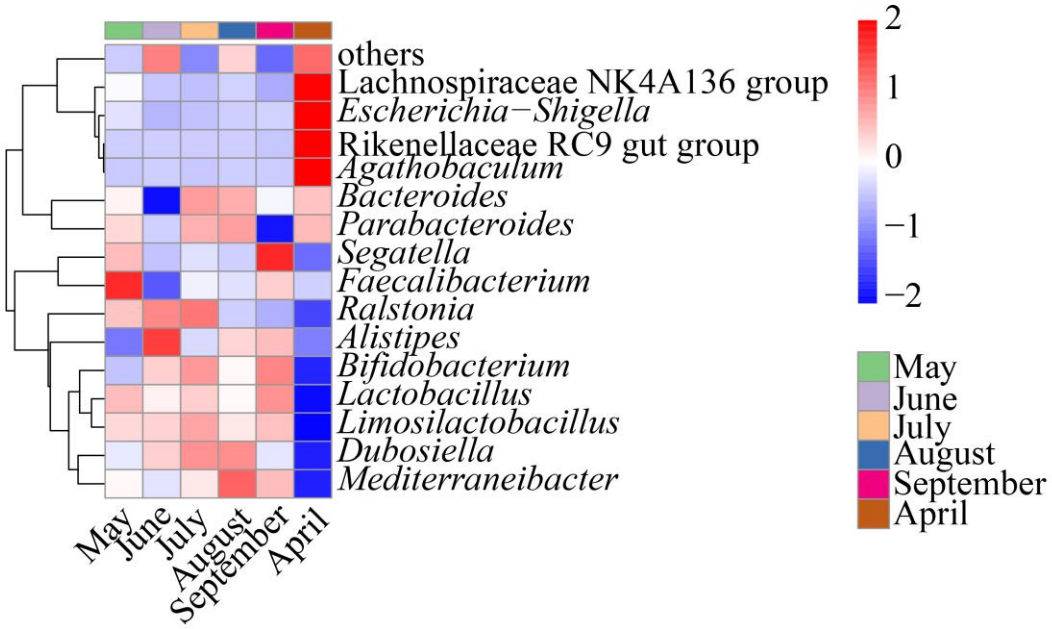

Figure S5 Heatmap of dominant taxa at the genus level across the larval stage of *C. beijingensis*. The color scale represents relative abundance, ranging from blue (low abundance) to red (high abundance).
