# Supplementary material for: Seasonal Dynamics Without Reset: Core Microbiota Stability Across Development in a Gall-Dwelling Weevil
Source: Insects. 2026 May 23;17(6):544. doi: 10.3390/insects17060544 (PMC13299049; doi:10.3390/insects17060544)
Supplement: Supplementary file 1 [file insects-17-00544-s001.zip › Table S.pdf]

Table S1 16S rRNA gene amplicon sequencing analysis of the symbiotic microbiota of *Coccotorus beijingensis*, including alpha and beta diversity assessments.

Sample ID	Raw Reads	Seqs Num	ASV Num	Total ASVs	Diversity indexes				
					Ace	Chao1	Shannon	Simpson	Coverage
S1	79944	75325	84	5473	85.0800	84.4333	0.5247	0.0941	1.0000
S2	79412	75192	68	5473	67.9483	69.2767	0.3934	0.0685	0.9999
S3	78658	73763	40	5473	40.2174	40.1000	0.3264	0.0636	1.0000
S4	78826	75128	48	5473	46.4318	48.1000	0.4715	0.1025	1.0000
S5	78095	74816	53	5473	53.0000	53.0000	0.4092	0.0727	1.0000
S6	78190	74132	46	5473	46.0000	46.0000	0.3025	0.0535	1.0000
S7	81279	63913	9	5473	NA	9.0000	0.1263	0.0245	1.0000
S8	78548	74117	73	5473	73.5981	73.1810	0.6853	0.1428	1.0000
b1	78213	73621	174	5473	174.5154	173.9200	0.5317	0.0815	1.0000
b2	78448	73260	177	5473	177.1755	176.9063	0.6831	0.1131	0.9999
b3	78934	74041	173	5473	174.1476	172.9917	0.5847	0.0926	1.0000
b4	79670	75236	166	5473	166.3477	165.9417	0.4676	0.0716	1.0000
b5	79206	73538	203	5473	201.7671	202.9783	0.7162	0.1134	1.0000
b6	79794	74111	126	5473	125.3154	126.4333	0.4298	0.0676	1.0000
b7	79743	71677	83	5473	83.1681	83.0000	0.3622	0.0589	1.0000
b8	79357	74429	195	5473	196.0601	194.8310	0.5443	0.0833	1.0000
b9	78235	73119	162	5473	162.7149	162.0875	0.5350	0.0845	1.0000
b10	79602	74055	153	5473	152.8562	153.2593	0.4733	0.0739	1.0000
A1	79184	68563	83	5473	83.1993	83.0000	1.4716	0.4607	1.0000
A2	78895	73425	105	5473	104.6106	105.1117	0.3736	0.0603	1.0000
A3	77932	70633	64	5473	64.1538	64.0000	0.3012	0.0503	1.0000
A4	80220	76004	190	5473	187.9984	189.9808	0.6044	0.0948	1.0000
A5	78075	73243	171	5473	171.1517	171.0000	1.1212	0.2819	1.0000
A6	79023	73727	140	5473	138.5239	140.4700	1.2407	0.4208	1.0000
A7	78113	72872	207	5473	207.1436	207.0000	0.8327	0.1318	1.0000
A8	78288	74129	132	5473	132.1773	132.1200	0.4602	0.0740	1.0000
A9	78704	72847	218	5473	217.1424	217.9000	1.0113	0.1637	1.0000
A10	79863	74740	162	5473	161.1673	162.2867	0.7030	0.1173	1.0000
B1	78069	70683	66	5473	66.0000	66.0000	0.3756	0.0651	1.0000
B2	79886	75821	187	5473	186.5903	186.9083	0.7769	0.1261	1.0000
B3	78512	73683	203	5473	203.0000	203.0000	1.1908	0.1977	1.0000
B4	78038	72987	221	5473	221.1426	220.9000	1.0791	0.1732	1.0000
B5	80700	75497	264	5473	263.0000	264.0000	1.6079	0.2609	1.0000
B6	78727	73695	215	5473	214.2905	215.6000	0.9607	0.1546	1.0000
B7	79343	67720	258	5473	257.4747	258.0333	3.2774	0.6544	1.0000
B8	81149	75697	161	5473	161.0000	161.0000	0.8249	0.1381	1.0000
B9	81015	70666	48	5473	48.0000	48.0000	0.3287	0.0567	1.0000
B10	80350	76135	194	5473	193.2985	193.9333	0.7512	0.1206	1.0000

C1	78354	73510	218	5473	218.0000	218.0000	1.4484	0.2352	1.0000
C2	78682	73916	252	5473	253.0000	252.1126	1.3669	0.2211	1.0000
C3	80479	75785	232	5473	232.6380	232.0000	1.3294	0.2162	1.0000
C4	80660	75937	183	5473	183.1395	183.1000	0.8105	0.1336	1.0000
C5	80282	74153	287	5473	285.2779	287.7533	1.7885	0.2891	1.0000
C6	80386	74925	97	5473	97.3761	97.0333	0.4049	0.0666	1.0000
C7	78150	68880	94	5473	94.0000	94.0000	0.6697	0.1159	1.0000
C8	80272	76320	199	5473	199.1511	199.0000	0.8722	0.1413	1.0000
C9	78932	74380	194	5473	194.2852	194.0000	0.8794	0.1419	1.0000
C10	80287	75493	230	5473	230.9163	229.7117	1.0136	0.1617	1.0000
D1	78993	74569	190	5473	190.5345	189.8250	0.6355	0.0990	1.0000
D2	78318	74643	159	5473	158.1682	159.0000	0.5441	0.0846	1.0000
D3	79612	74648	176	5473	176.3305	176.0000	0.6401	0.1028	1.0000
D4	79224	74432	99	5473	99.4104	99.2417	0.3633	0.0591	1.0000
D5	77944	68213	16	5473	16.0000	16.0000	0.1389	0.0257	1.0000
D6	79007	75037	215	5473	216.0026	215.0619	0.7159	0.1112	1.0000
D7	80643	76110	93	5473	93.1755	93.0000	0.3504	0.0573	1.0000
D8	79383	75316	127	5473	127.0000	127.1117	0.4177	0.0668	1.0000
D9	80211	73170	65	5473	65.0000	65.0000	0.2990	0.0492	1.0000
D10	78530	74189	154	5473	154.3205	153.8667	0.5917	0.0950	1.0000

Table S2 Amplicon sequence variant (ASV) counts per sample of *Coccotorus beijingensis* across six taxonomic levels.

Sample ID	Phylum	Class	Order	Family	Genus	Species
S1	6	9	25	38	47	45
S2	5	7	19	27	37	36
S3	4	6	17	20	24	25
S4	4	6	17	24	27	26
S5	5	7	16	24	25	29
S6	6	8	21	28	31	30
S7	4	5	5	6	6	6
S8	5	9	25	37	42	43
b1	9	16	36	53	75	67
b2	10	16	38	54	70	64
b3	10	15	34	45	56	74
b4	10	15	36	55	68	71
b5	11	18	37	53	74	84
b6	9	13	27	41	47	47
b7	7	10	22	29	39	37
b8	10	14	36	50	70	76
b9	9	12	32	42	60	62
b10	10	16	31	42	58	58
A1	7	10	17	26	34	40
A2	7	12	26	38	50	50
A3	5	9	20	28	33	31
A4	10	15	38	52	75	81
A5	9	16	34	49	66	66
A6	8	12	32	43	59	61
A7	11	19	40	54	69	72
A8	9	15	31	42	58	63
A9	8	12	32	45	74	78
A10	12	17	36	51	66	64
B1	5	9	20	30	35	34
B2	10	16	40	56	70	70
B3	11	15	40	51	66	72
B4	12	20	40	51	81	83
B5	10	19	35	49	72	77
B6	9	14	36	52	79	85
B7	11	17	36	48	72	75
B8	9	15	35	50	64	59
B9	6	8	18	24	26	24
B10	10	16	36	52	71	86
C1	11	17	46	66	85	90

C2	9	16	39	53	76	88
C3	9	13	40	55	78	95
C4	12	16	39	56	70	72
C5	9	15	36	48	82	103
C6	8	13	30	36	45	39
C7	8	11	20	31	43	38
C8	13	19	39	54	72	83
C9	10	14	39	54	67	68
C10	10	19	41	55	72	78
D1	8	15	31	46	71	78
D2	12	19	40	51	68	59
D3	9	16	37	50	65	66
D4	8	14	30	41	49	49
D5	4	5	8	11	11	9
D6	9	14	38	51	76	77
D7	8	13	25	35	43	35
D8	8	13	28	40	54	51
D9	5	8	15	24	30	30
D10	8	14	31	46	56	68

Table S3 Pairwise PERMANOVA tests (using the adonis2 function in R) on beta-diversity distance matrices of the symbiotic microbiota of *Coccotorus beijingensis*.

Pairwise comparison	Unweighted unifrac		Bray curtis	
	P	R^2	P	R^2
May vs June	0.691	0.0497	0.013	0.0699
May vs July	0.147	0.0574	0.0048	0.0749
May vs August	0.122	0.0585	0.0038	0.0749
May vs September	0.037	0.0623	0.001	0.0971
May vs April	0.001	0.1898	0.001	0.2349
June vs July	0.178	0.0581	0.836	0.0479
June vs August	0.05	0.0643	0.011	0.0675
June vs September	0.395	0.0533	0.007	0.0719
June vs April	0.001	0.1722	0.001	0.2487
July vs August	0.666	0.0501	0.502	0.0521
July vs September	0.065	0.0618	0.114	0.0616
July vs April	0.001	0.1843	0.001	0.2648
August vs September	0.013	0.0675	0.054	0.0654
August vs April	0.001	0.2062	0.001	0.2585
September vs April	0.002	0.1633	0.001	0.2813

Note: PERMANOVA (adonis2) tests were conducted on beta-diversity distance matrices. The R^2 statistic represents the proportion of variance in bacterial community composition explained by the grouping factor.
